# What Is Specific and What Is Shared Between Numbers and Words?

**DOI:** 10.3389/fpsyg.2016.00022

**Published:** 2016-02-02

**Authors:** Júlia B. Lopes-Silva, Ricardo Moura, Annelise Júlio-Costa, Guilherme Wood, Jerusa F. Salles, Vitor G. Haase

**Affiliations:** ^1^Developmental Neuropsychology Laboratory, Department of Psychology, Universidade Federal de Minas GeraisBelo Horizonte, Brazil; ^2^Department of Neuropsychology, Institute of Psychology, Karl-Franzens University of GrazGraz, Austria; ^3^Núcleo de Estudos em Neuropsicologia Cognitiva, Institute of Psychology, Universidade Federal do Rio Grande do SulPorto Alegre, Brazil

**Keywords:** reading, spelling, number transcoding, phonemic awareness, intelligence

## Abstract

Reading and spelling performance have a significant correlation with number transcoding, which is the ability to establish a relationship between the verbal and Arabic representations of numbers, when a conversion of numerical symbols from one notation to the other is necessary. The aim of the present study is to reveal shared and non-shared mechanisms involved in reading and writing of words and Arabic numerals in Brazilian school-aged children. One hundred and seventy-two children from second to fourth grades were evaluated. All of them had normal intelligence. We conducted a series of hierarchical regression models using scores on word spelling and reading single words and Arabic numerals, as dependent variables. As predictor variables we investigated intelligence, the phonological and visuospatial components of working memory (WM) and phonemic awareness. All of the writing and reading tasks (single word spelling and reading as well as number reading and number writing) were significantly correlated to each other. In the regression models, phonological WM was specifically associated to word reading. Phonemic awareness was the only cognitive variable that systematically predicted all of the school skills investigated, both numerical and word tasks. This suggests that phonemic awareness is a modular cognitive ability shared by several school tasks and might be an important factor associated to the comorbidity between dyslexia and dyscalculia.

## Introduction

Reading and math performance in school-aged children are related in important ways. In this study, we set out to investigate shared and non-shared mechanisms involved in word reading/spelling and number transcoding abilities. Genetically informed studies indicate that performance in standardized math, spelling, and reading achievement tests substantially correlate at the phenotypic and genetic levels, both in typically and atypically developing children (Kovas et al., 2005, 2007; Hart et al., 2009). The discovery of genetic correlations between reading/spelling and math achievement led to the formulation of the “generalist genes” hypothesis, according to which both kinds of academic abilities share multifactorial genetic and environmental etiologies across levels of performance (Plomin and Kovas, 2005). Comorbidity rates are high between multifactorial developmental dyslexia and dyscalculia (Landerl and Moll, 2010). These data suggest that reading and mathematical abilities may depend on common cognitive mechanisms. Evidence for shared mechanisms is so overwhelming that the DSM-5 Task Force considered abolishing the distinction between specific reading and math learning disorders (Tannock, 2013).

Other evidence points to dissociations between reading and math performance. Pure cases of multifactorial dyslexia and dyscalculia hint at non-shared specific cognitive mechanisms that may characterize distinct entities or subtypes (Rubinsten and Henik, 2006; Tressoldi et al., 2007; Landerl et al., 2009; Haase et al., 2014). Analysis of family recurrence patterns in developmental dyslexia and dyscalculia indicate that besides common genetic factors, also specific ones may be involved. For example, Landerl and Moll (2010) observed cross-condition family recurrence, but recurrence rates were higher in dyslexia in families of reading disabled individuals and higher in dyscalculia in families of math disabled individuals.

Shared and non-shared mechanisms can be identified at the neural level (Ashkenazi et al., 2013). The neural networks involved in word reading and arithmetic learning are only partially overlapping. Learning of word reading has been shown to depend on a neural network consisting, among other areas, of the inferior lateral occipito-temporal cortex, posterior superior temporal cortex and adjacent inferior parietal areas, and lateral inferior prefrontal cortex (Dehaene, 2009; Ashkenazi et al., 2013). Learning of arithmetics is associated to the structural and functional integrity of a frontoparietal network converging on the intraparietal sulcus, but also including inferior lateral occipito-temporal cortex, inferior parietal cortex, and hippocampus (Kaufmann et al., 2011; Moeller et al., 2011; Ashkenazi et al., 2013; Matejko and Ansari, 2015).

In a systematic review, Ashkenazi et al. (2013) identified both non-shared and shared neural underpinnings of reading and math learning. Word reading learning depends on the structural-functional integrity of the left hemisphere while math learning relies more heavily, but not exclusively, on right hemisphere mechanisms. Different parietal areas are also involved. Reading depends more on the inferior parietal cortex (supramarginal gyrus, angular gyrus) and math on the intraparietal sulcus. Overlapping neural components are situated at the lateral inferior occipito-temporal cortex, inferior parietal cortex (supramarginal gyrus, angular gyrus) and inferior frontal gyrus.

Which are the possible shared and non-shared mechanisms at the cognitive level of description? In order to search for an answer to this question, it is important to investigate outcome measures shared by reading and arithmetics. Standardized achievement tests are not good indicators because they evaluate abilities at different complexity levels, recruiting semantic and reasoning processes in different degrees. We focused then on some relatively low-level abilities of word reading/spelling and basic numerical processing (Arabic number reading and Arabic number writing).

These basic, decontextualized and less semantically loaded abilities have been consistently implicated in reading and math performance both in typical and atypical populations. In the case of reading, for example, it has been assumed that reading at the word level is an important precursor of more advanced mechanisms of reading comprehension (Gough, 1996; Florit and Cain, 2011). The same can be argued for arithmetics: basic knowledge and processing of verbal and Arabic numerals is an important precursor of later arithmetics learning (Moeller et al., 2011), as well as a marker of math learning difficulties (Moura et al., 2013, 2015).

Focusing on the basic abilities at the lexical and numerical processing levels, we also hope to uncover meaningful patterns of association and dissociation between cognitive variables and reading/spelling- and math-related performances in early school age. According to Ashkenazi et al. (2013), there are three main sets of variables which are associated to reading/spelling and arithmetics performance: (a) phonological processing, mainly at the phonemic level, and including rapid access to phonological representations (RAN tasks), phonological short-term memory, and phonemic awareness, is consistently associated to word reading learning (Castles and Coltheart, 2004; Melby-Lervåg et al., 2012); (b) numerical magnitude representations (Landerl et al., 2004; Halberda et al., 2008; Piazza et al., 2010; Mazzocco et al., 2011; Pinheiro-Chagas et al., 2014) or symbolic access to magnitudes (Rubinsten and Henik, 2005; Rousselle and Noël, 2007, see review in De Smedt et al., 2013) have been proposed as mechanisms underlying math learning and its difficulties; and (c) working memory (WM) is relevant for both reading/spelling and math learning (Swanson et al., 2006; Peng and Fuchs, 2014). The question is whether the verbal and non-verbal aspects of WM differentially affect reading/spelling and math performance (Peng and Fuchs, 2014).

The picture is, however, complicated by the fact that arithmetics-related abilities are more heterogeneous than reading-related abilities. Research increasingly shows that some aspects of arithmetics and number processing may be dependent on verbal processes, sharing mechanisms with reading learning and dyslexia (Simmons and Singleton, 2008; De Smedt and Boets, 2010) while others rely on non-symbolic magnitude processing (Landerl et al., 2004; Halberda et al., 2008; Piazza et al., 2010; Mazzocco et al., 2011; Pinheiro-Chagas et al., 2014). For instance, phonemic awareness has been identified as a predictor of not only reading but also math abilities (Hecht et al., 2001). Foremost, among the numerical abilities dependent on verbal processes are number transcoding (Lopes-Silva et al., 2014), retrieval of arithmetic facts (De Smedt and Boets, 2010), and word problem solving (Jordan et al., 2003; Powell et al., 2009). Phonological representations have also been included as an important component in some models of numerical transcoding, such as ADAPT (Barrouillet et al., 2004).

Following Ashkenazi et al. (2013), we identify intelligence, WM (both phonological and visuospatial) and phonemic awareness as cognitive dimensions relevant to acquiring early reading/spelling and math abilities. We will discuss the role of these general and specific abilities in word reading/spelling and numerical transcoding.

Intelligence is an important long-term predictor of school achievement assessed with standardized omnibus tests (Deary et al., 2007). One could ask if intelligence would not only be associated with achievement at higher and more complex levels of performance. Correlations with intelligence are higher for reading comprehension than for word decoding (Nobre and Salles, 2014) and a large body of literature also implicates intelligence in the acquisition of early visual word decoding skills (Stanovich et al., 1984; Tunmer and Nesdale, 1985; Juel et al., 1986). The association of intelligence and word reading achievement is also observed in the intellectually disabled population (Levy et al., 2002).

The role of intelligence in number reading and writing has been the focus of less research. However, extant data indicate that general cognitive ability is significantly associated with Arabic number reading and dictation in the school-age population (Moura et al., 2013; Lopes-Silva et al., 2014). Probably, intelligence is important for learning words and number reading and writing skills, because at the age children are involved with these tasks, this represents quite an accomplishment, a novel and challenging task that mobilizes their best available cognitive resources.

Regarding visuospatial WM, Zuber et al. (2009) verified a specific association between Corsi blocks and a number writing task in 7-year-old German speaking children. As it is known, the verbal numeric notation in the German language is characterized by the inversion of units and decades in two-digit numbers. The authors observed that over 50% of the errors made by children had a syntactic nature, involving the inversion of units and decades. These results were confirmed by Pixner et al. (2011) in Czech speaking children, a language that uses both direct and inverted systems for naming two-digit numbers. They showed that Czech children committed more syntactic errors when they wrote numbers in the inverted form and these errors were associated to visuospatial WM. Furthermore, Moura et al. (2013) observed that the Corsi Blocks task had a moderate correlation with a number writing task and a weak, but significant, correlation with a number reading task.

The association between visuospatial WM and reading/spelling is unclear and more studies are required. A recent investigation explored the complete WM profile of children with poor reading ability (Dawes et al., 2015). Results showed that these children had low performance in the phonological loop and central executive components, but typical abilities in the visuospatial sketchpad. Additionally, a study with school-aged children with and without reading difficulties revealed no influence of visuospatial WM on reading development (Cormier and Dea, 1997).

Phonological WM is the mechanism involved in the temporary storage and manipulation of verbal items (Baddeley, 2007). Concerning the development of mathematical abilities, it has been argued that phonological WM is recruited in basic skills, such as counting (Noël, 2009) and in calculations based on procedural strategies (Hecht, 2002; McKenzie et al., 2003; Imbo and Vandierendonck, 2007), but not on fact retrieval (Seyler et al., 2003). Furthermore, phonological WM is also predictive of later mathematics achievement, as discussed above. Phonological WM skills have also been consistently related to single word reading performance (Leather and Henry, 1994; Oakhill and Kyle, 2000; Alloway et al., 2005). A recent meta-analysis showed persistent deficits in phonological WM in children with reading disability, independently of chronological age and intelligence (Kudo et al., 2015).

Phonological awareness can be investigated by means of tasks that demand the distinction between the sounds that constitute words, such as rhyme detection and blending isolated sounds to create words (Lewkowicz, 1980). Recently, Cunningham et al. (2015) investigated how the nature of the phonological task and (a) the linguistic nature of the stimuli, (b) the phonological complexity of the stimuli, and (c) the production of a verbal response, can influence the relationship between the task and reading. They have argued that the production of a verbal response is important for the task to be a good predictor of decoding. In this study, we will use a phoneme elision task, in which the child is required to say what a word would be after deleting a certain phoneme and, furthermore, the child should verbally emit the response.

A question that demands further discussion lies in the fact that the phonological complexity of the stimulus might be a confounding factor to mask the association to reading (Cunningham et al., 2015). One important hypothesis is that phonological WM may act as a mediator between phonological awareness and reading performance, as measures of phonological awareness generally involve phonological WM resources (Dufva et al., 2001). In the specific case of phoneme elision, there is the need of a conscious access to phonological representations and this may lead to special demands on access mechanisms (Ramus and Szenkovits, 2008). Furthermore, the importance of phonemic awareness in reading/spelling and math skills might be overrated due to phonological WM influences which would then support the hypothesis of an access deficit in dyslexia (Boets et al., 2013). It is important to simultaneously investigate the influence of both variables to have more information on the specific correlates of each of them.

Children with developmental dyslexia, who perform poorly on phonological processing tasks, such as phonemic awareness, frequently exhibit deficits in mathematics. According to the weak phonological representation hypothesis (Simmons and Singleton, 2008), phonological processing deficits would impair aspects of mathematical cognition that involve the manipulation of verbal codes, but not those that are not verbally coded, such as approximate addition and non-symbolic magnitude comparison. Nevertheless, only few studies (see, Michalczyk et al., 2013; Lopes-Silva et al., 2014) have investigated the association between phonological processing and number transcoding.

The aim of the present study is to investigate shared and non-shared mechanisms involved in reading and writing words and Arabic numerals in school-aged children. Our main goal was to disentangle the role of phonemic awareness and its impact on lexical and numerical tasks controlling for cognitive variables which may have an important impact. We hypothesize that even after controlling for the influence of intelligence and broader reading and writing skills, phonemic awareness would be an important predictor since all of these tasks involve some sort of verbal processing. In order to investigate that, we conducted a series of hierarchical regression models using scores of reading and writing of single words and Arabic numbers tasks, as dependent variables. As predictor variables we investigated intelligence, the phonological and visuospatial components of WM and phonemic awareness skills.

## Materials and Methods

The study was approved by the local research ethics committee (COEP–UFMG). Children participated only after informed consent was obtained. It was obtained in written form from parents and orally from children.

### Participants

We have assessed 207 children from second to fourth grades of public schools from Belo Horizonte, Brazil. Data collection took place in the participants’ schools. We excluded one child who did not complete the entire battery, four children due to low intelligence (performance on Raven’s Colored Progressive Matrices below one standard deviation from the mean), and 30 children were excluded from further analysis, because either they had a poor *R*^2^ on the fitting procedure to calculate their internal Weber fraction on the non-symbolic comparison task or their Weber Fraction exceeded the limit of discriminability of our task (*w* > 0.6). The final sample was constituted by 172 children with ages ranging from 7 to 11 years (mean = 8.86[0.96] years), 55.2% girls.

### Instruments

At first, the intelligence (Raven’s CPM), word spelling (Brazilian School Achievement Test – TDE) and number transcoding (Number writing task) were evaluated in small groups of approximately nine children. Subsequently, we tested number transcoding (Number reading task), word reading (reading subtest of Brazilian School Achievement Test – TDE), phonemic awareness (Phoneme Elision), and WM (Corsi Blocks and Digit Span).

#### (a) Raven’s Colored Progressive Matrices

General intelligence was assessed with the age-appropriate Brazilian validated version of Raven’s Colored Matrices (Angelini et al., 1999).

#### (b) Brazilian School Achievement Test

The Teste de Desempenho Escolar (TDE, Stein, 1994) is the most widely used standardized test of school achievement in Brazil. The TDE comprises three subtests: arithmetics, single-word spelling, and single-word reading. Norms are provided for school-aged children between first and sixth grades. The arithmetics subtest is composed of three simple orally presented word problems and 35 written arithmetic calculations of increasing complexity. The spelling subtest consists of dictation of 34 words of increasing syllabic complexity. The single-word reading subtest of the TDE consists of 70 stimuli, which must be read aloud by the individual participant. Reliability coefficients (Cronbach α) are 0.87 or higher. Children are instructed to work as hard as they can, without time limits. In the present study we used the data of reading and spelling subtests, only.

#### (c) Arabic Number Writing

To evaluate number transcoding, children were instructed to write the Arabic forms of dictated numbers. This task consists of 40 items, up to four digits (3 one-digit numbers, 9 two-digit numbers, 10 three-digit numbers, and 18 four-digit numbers). The one- and two-digit numbers were classified as “lexical items” (12 items), and the other 28 items require the use of algorithm-based rules in order to be written (Barrouillet et al., 2004; Camos, 2008). As shown in previous investigations, this task presents high internal consistency (*KR* – 20 = 0.96; Moura et al., 2013, 2015; Lopes-Silva et al., 2014).

#### (d) Arabic Number Reading

A total of 28 Arabic numbers with one to four digits were printed in a booklet and presented to children one at a time. Children were instructed to read them aloud. Items were grouped into three categories according to their complexity, indexed by the number of transcoding rules established by the ADAPT model. The three- and four-digit numbers were chosen to avoid presenting numbers with very strong lexical entries and to maintain the focus on syntactic complexity. This task has been used in previous studies, and the consistency of this task was *KR* – 20 = 0.92 (Moura et al., 2013).

#### (e) Phoneme Elision

This is a widely accepted measure of phonemic awareness (Wagner and Torgesen, 1987; Castles and Coltheart, 2004; Melby-Lervåg et al., 2012). The child hears a word and must say what the word would be if a specified phoneme in the word were to be deleted (e.g., “filha” without /f/ is “ilha” [in English, it would be similar to “cup” without /k/ is “up”). The test comprises 28 items: in eight items, the child must delete a vowel, and in the other 20, a consonant. The consonants to be suppressed varied by place and manner of articulation. The phoneme to be suppressed could be in different positions within the words, which ranged from two to three syllables. This task has been used in a previous study with a comparable sample (Lopes-Silva et al., 2014), and the internal consistency of the task is 0.92 (*KR* – 20 formula).

#### (f) Corsi Blocks

This test is a measure of the visuospatial component of WM. It is constituted by a set of nine blocks which are tapped, in a certain sequence, by the examiner. The test starts with sequences of two blocks and can reach a maximum of nine blocks. We used the forward and backward orders according to Kessels et al. (2000). In the forward condition, the child is instructed to tap the blocks on the same order as the examiner, in the backward condition, in the inverse order. We evaluated the total score (correct trials × span) in the backward order.

#### (g) Digit Span

Verbal short-term memory was assessed with the Brazilian WISC-III Digits subtest (Figueiredo, 2002). Performance in the forward order was considered a measure of phonological short-term memory, and the backward order was used to assess verbal WM. We also evaluated the total scores of the backward order.

## Results

Raw scores were *z*-transformed by school grade. By doing so, we aimed at controlling for any possible educational influence on children’s performance. We operationalized the investigation of phonemic awareness using a phoneme elision task, and verbal and visuospatial WM were investigated with Digit Span and Corsi Blocks, respectively. At first, we investigated the association between the tasks using Pearson’s correlations. Afterwards, we performed four hierarchical regression models with each of these reading and writing skills as dependent variables to investigate which cognitive abilities would predict their performance. In order to control for the shared variance among numerical and verbal tasks, whenever one of these tasks was set as the dependent variable, the other was inserted in the first step of the regression model, using the enter method (i.e., when number reading task was the dependent variable, word reading was included as an independent variable in the first step of the model). We also included intelligence in the first step of these models. In the second step, phonological and visuospatial WM and phonemic awareness were included as predictors. The stepwise method was used in this second step to avoid redundancy and to guarantee a high degree of parsimony.

### Associations Between Cognitive Variables and Reading and Writing Processes

To explore the general pattern of association between the cognitive variables and reading and writing of numbers and words, we investigated the correlations between them (**Table 1**).

**Table 1:** Correlations between the neuropsychological measures.

	Word spelling	Word reading	Number writing	Number reading	Phoneme elision	Digit span	Corsi blocks
Raven	0.449^∗∗^	0.313^∗∗^	0.401^∗∗^	0.411^∗∗^	0.371^∗∗^	0.359^∗∗^	0.329^∗∗^
Word spelling	1	0.719^∗∗^	0.593^∗∗^	0.562^∗∗^	0.557^∗∗^	0.317^∗∗^	0.155^∗^
Word reading		1	0.503^∗∗^	0.598^∗∗^	0.693^∗∗^	0.395^∗∗^	0.149
Number writing			1	0.762^∗∗^	0.483^∗∗^	0.271^∗∗^	0.244^∗∗^
Number reading				1	0.602^∗∗^	0.287^∗∗^	0.238^∗∗^
Phoneme elision					1	0.366^∗∗^	0.253^∗∗^
Digit span						1	0.354^∗∗^
Corsi blocks							1

*^∗^Correlation is significant at the 0.05 level (2-tailed). ^∗∗^Correlation is significant at the 0.01 level (2-tailed).*

As can be seen in **Table 1**, all of the writing and reading tasks (TDE word reading and spelling, as well as number reading and number writing) were significantly correlated (all *r*’s > 0.55, *p* < 0.001). Word reading and word spelling were highly correlated to each other, as well as number reading and number writing. Intelligence also presented correlations between 0.30 and 0.40 with all of the variables. Both phonological processing tasks correlated with the verbal and numerical ones. Correlations for phoneme elision were in the 0.37–0.69 range, and correlations for digit span were in the 0.27–0.39 range. Corsi Blocks did not present significant correlations to the word reading task.

### Specific Predictors of Reading and Writing Skills

To further explore the association between these variables and to have a more fined-grained perspective regarding the predictive power of them on each of the verbal and numerical skills, we conducted separate hierarchical regression models.

We calculated regression models including intelligence and the analogous variables in the first step and the other cognitive variables in the second one. By doing so, we aimed at investigating the pattern of association between these variables, once we had controlled for more general cognitive skills. Variance for each kind of number task (e.g., reading or writing) was predicted by the homologous tasks with words (e.g., reading or writing) and vice-versa (*r*^2^ ranging from 0.36 to 0.41) and by phoneme elision (*r*^2^ ranging from 0.03 to 0.17). As can be seen in **Tables 2–5**, phoneme elision was the only task that was a significant predictor in the four hierarchical models. It is important to note that phonological WM was associated to word reading, but its influence was much smaller compared to phonemic awareness.

**Table 2:** Regression analysis for number reading (adjusted *r*^2^ = 0.449).

Predictor	Beta	Partial *t*	Significance	*r*^2^ change
Intercept		–2.387	0.018	
Raven	0.196	3.194	0.002	
Word reading	0.327	4.142	<0.001	0.413
Digit span	–0.03	–0.469	0.64	Excluded
Corsi blocks	0.055	0.904	0.367	Excluded
Phoneme elision	0.303	3.747	<0.001	0.045

**Table 3:** Regression analysis for word reading (adjusted *r*^2^ = 0.539).

Predictor	Beta	Partial *t*	Significance	*r*^2^ change
Intercept		0.441	0.660	
Raven	–0.034	–0.575	0.566	
Number reading	0.279	4.143	<0.001	0.363
Digit span	0.151	2.614	0.010	0.018
Corsi blocks	–0.099	–1.746	0.083	Excluded
Phoneme elision	0.482	7.136	<0.001	0.168

**Table 4:** Regression analysis for number writing (adjusted *r*^2^ = 0.390).

Predictor	Beta	Partial *t*	Significance	*r*^2^ change
Intercept		–5.357	<0.001	
Raven	0.138	2.042	0.043	
Word spelling	0.421	5.557	<0.001	0.374
Digit span	0.019	0.291	0.804	Excluded
Corsi blocks	0.096	1.509	0.867	Excluded
Phoneme elision	0.197	2.71	0.007	0.026

**Table 5:** Regression analysis for word spelling (adjusted *r*^2^ = 0.465).

Predictor	Beta	Partial *t*	Significance	*r*^2^ change
Intercept		4.421	<0.001	
Raven	0.187	2.984	0.003	
Number writing	0.369	5.557	<0.001	0.405
Digit span	0.046	0.737	0.462	Excluded
Corsi blocks	–0.087	–1.454	0.148	Excluded
Phoneme elision	0.309	4.721	<0.001	0.070

## Discussion

In the present study, we investigated the cognitive variables that underlie the performance of reading and writing skills for both numbers and words in a sample of Brazilian school-aged children. Our results can be summarized into two mains topics: the specific influence of phonemic awareness on reading and writing words and numbers and the impact of non-verbal intelligence on them. We have found a prominent role of phonemic awareness which was consistently associated to all the reading and writing modalities we have assessed. As far as we know, this is the first study to simultaneously investigate these four skills and the cognitive variables related to them.

### Phonemic Awareness as a Common Mechanism Shared Between Reading and Writing of Both Numbers and Words

Phonemic awareness is an important underlying factor of reading acquisition (Ehri et al., 2001) and deficits in it are associated to reading disabilities and dyslexia (Lyon et al., 2003). A puzzling aspect of this association lies in the fact that there is a reciprocal relationship between phonological processing and reading skills: when children begin to read, their reading skills become the best predictor of their own reading development (Bell et al., 2003). In this study, we aimed at controlling this confounding variable to be able to analyze the interplay between numbers and words. To do so we investigated the influence of cognitive variables in word reading, for example, by controlling for the impact of number reading. We have found a consistent role of phonemic awareness in both number and word reading and writing, after controlling for other cognitive variables.

Even though the relation of phonemic awareness and word reading skills is well documented in the literature (Vellutino et al., 2004), the association between phonemic awareness and word spelling is less robust. Nevertheless, the interaction between phonological processing and other cognitive skills, such as syntactic awareness and naming-speed, are taken as evidence in favor of an integrative hypothesis (Plaza and Cohen, 2003). In our study, we have found that phonological WM was an important predictor of single word reading but not spelling, A possible reason for that lies in the fact that in the spelling subtest of Brazilian School Achievement Test, children hear the word three times: first the examiner dictates only the word itself, afterward inside a sentence, and then the isolated word again. Since the child hears the word so many times, other variables that are associated to spelling skills, such as orthographic rules, may play a more important role and this should be investigated in further studies. Despite this, reading and spelling are highly correlated [0.71 in our study and from 0.77 to 0.86 according to Ehri (1997)] and both have phonemic awareness as a common underlying mechanism.

Regarding number transcoding and phonological processing skills, there is even less investigation. Even though mathematical and reading/spelling disabilities have a high comorbidity rate (Landerl and Moll, 2010) there are not so many studies that deeply investigate what is shared in this most basic level: single word and number writing and reading. One plausible argument to explain the comorbidity is the weak phonological representation hypothesis which explicitly states that any aspect of numerical cognition that is associated to a verbal code would be impaired in dyslexics, since they would have fuzzier phonological representations. According to the ADAPT model of number writing, one of the first steps in the verbal to Arabic number transcoding is phonological encoding (Barrouillet et al., 2004). Phonemic awareness might be a distal source of influence on this phonological step in the model. Regarding number reading, the picture is less clear: the procedural steps between Arabic to verbal number transcoding have been described in terms of intermediary semantic representations which can have a verbal-linguistic component (Power and Dal Martello, 1990). Nevertheless, cognitive models of number reading do not usually take linguistic processes into consideration. One can assume, however, that phonemic awareness might also be an important linguistic mechanism associated to this modality of transcoding.

The association between spelling and word reading to mathematics disabilities depends on the cutoff criteria used to define them. Most studies have investigate associations between word reading and arithmetic, especially in dyslexic samples (Boets and De Smedt, 2010; Göbel and Snowling, 2010). Landerl and Moll (2010) investigated the comorbidity rates between Spelling Disabilities (SD); Reading Disabilities (RD), and Arithmetic Disabilities (AD) in a large population-based sample of elementary school children. They reported an interesting finding: the rate of comorbidity between AD and RD decreases when children are defined based on a more stringent criteria, whereas the rate between arithmetics and spelling remains constant. The interplay between reading and writing of numbers and words is rather complex and one should always have in mind that it depends on the measures and criteria used. It is also important to emphasize that the power of phonemic awareness as an indicative of literacy skills changes according to grade (Moll and Landerl, 2009): our results should be cautiously interpreted and circumscribed to Portuguese-speakers who are on the second to fourth grades.

### The Contribution of Intelligence to Reading and Writing

The use of intelligence as a covariate in studies of children with learning disabilities has been criticized (Dennis et al., 2009). According to these authors, the high correlation of IQ and school performance is underspecified, as intelligence is both a predictor and an outcome variable. The dual role of IQ and its correlation with school achievement increases the risk of statistical distortions caused by regression to the mean. According to this line of reasoning, including IQ-related measures as covariates is not only irrelevant but also an improper conduct.

Another line of argumentation is also defensible. Estimates of general cognitive ability have been repeatedly found to be among the best predictors of school achievement and other psychosocial outcomes (Strenze, 2007; Deary and Johnson, 2010). According to this perspective, not all IQ-related measures are equally influenced by educational experience and socioeconomic status. Some measures, such as vocabulary, are heavily dependent on educational and reading experience, while others such as the Raven’s CPM are more related to fluid general intelligence (Carpenter et al., 1990) and are less dependent on educational experience. The fluid Intelligence (Raven’s CPM) is closely related with WM in childhood and this relation is primarily explained by the executive component of WM (Sbicigo et al., 2014).

Mastery of word and number processing at the lexical level is an enormous task for children at early school age. There is evidence, for example, that mastery over Arabic number dictation is reached only after 3–4 years of schooling in typically developing children (Moura et al., 2013, 2015). Associations between general cognitive ability and reading are higher for reading comprehension (*r*^2^ = 0.44) than for word decoding (*r*^2^= 0.05, Shatil and Share, 2003). But general cognitive ability is not irrelevant for reading at the word level. This is corroborated by higher prefrontal activation levels in beginning readers than in older, more proficient, children and adults (Schlaggar et al., 2002; Turkeltaub et al., 2003). According to Mayes et al. (2008), IQ tests can predict academic achievement, but its predictive power is increased when phonological WM and visuo-motor integration are also included.

Early school abilities related to word and number reading and writing may depend on both domain-general and domain-specific cognitive abilities. Sources of influence are both shared and non-shared across codes and tasks. Phonological and visuospatial WM tasks could be uniquely associated, respectively, to verbal lexical and numerical tasks. But these effects disappear or are attenuated when general non-verbal intelligence is covaried (the zero-order correlation between number writing and digit span is *r* = 0.271; *p* < 0.001 and the partial correlation controlling for intelligence decreases to *r* = 0.149; *p* = 0.052). Phonemic awareness seems to represent a shared source of variance, common to both kinds of codes and tasks, exerting effects over and above general intelligence.

## Conclusion and Implications

These findings suggest that phonemic awareness may be considered as a domain-specific cognitive mechanism which is strongly associated to reading and writing of both numbers and words. From these results, one can infer that phonemic awareness is a mechanism shared by numerical and verbal domains, which might also be a candidate associated to the high comorbidity rate between dyslexia and dyscalculia. It is important to note that this specific mechanism is related to number and word reading and writing and should, therefore, be taken into account in intervention models.

## Author Contributions

All authors have contributed significantly and all of us agree with the content of the manuscript.

## Conflict of Interest Statement

The authors declare that the research was conducted in the absence of any commercial or financial relationships that could be construed as a potential conflict of interest.
